# Statistical and computational methods for integrating microbiome, host genomics, and metabolomics data

**DOI:** 10.7554/eLife.88956

**Published:** 2024-06-04

**Authors:** Rebecca A Deek, Siyuan Ma, James Lewis, Hongzhe Li

**Affiliations:** 1 Department of Biostatistics, University of Pittsburgh Pittsburgh United States; 2 Department of Biostatistics, Vanderbilt School of Medicine Nashville United States; 3 Division of Gastroenterology and Hepatology, Perelman School of Medicine, University of Pennsylvania Philadelphia United States; 4 Department of Biostatistics, Epidemiology, and Informatics, Perelman School of Medicine, University of Pennsylvania Philadelphia United States; University of California, San Francisco United States; University of Sydney Australia

**Keywords:** data integration, network analysis, causal inference, systems biology

## Abstract

Large-scale microbiome studies are progressively utilizing multiomics designs, which include the collection of microbiome samples together with host genomics and metabolomics data. Despite the increasing number of data sources, there remains a bottleneck in understanding the relationships between different data modalities due to the limited number of statistical and computational methods for analyzing such data. Furthermore, little is known about the portability of general methods to the metagenomic setting and few specialized techniques have been developed. In this review, we summarize and implement some of the commonly used methods. We apply these methods to real data sets where shotgun metagenomic sequencing and metabolomics data are available for microbiome multiomics data integration analysis. We compare results across methods, highlight strengths and limitations of each, and discuss areas where statistical and computational innovation is needed.

## Introduction

Epidemiological studies of the microbiome are increasingly adopting multiomics designs, including the collection of metagenomics, metatranscriptomics, metabolomics, proteomics, as well as host genetics and genomics data (Lloyd-Price et al., 2019; Dekkers et al., 2022; Tanes et al., 2021; Chen et al., 2022; Diener et al., 2022; Priya et al., 2022; Lötstedt et al., 2023; Long et al., 2020). Such studies provide rich information to link host health conditions to candidate molecular biomarkers, including microbial taxa, functional pathways, transcriptional activities, metabolic products, and the interplay thereof. To meet the advancement in data richness, both in available data modalities and in sheer sample size, statistical and computational methods for analyzing multiomics microbiome, host genomics, and metabolomics data have also been expanding. Such methods are often adapted from either existing groundwork in a single modality (e.g. microbial relative abundance analysis) or other data integration techniques in general multiomics molecular epidemiology.

Broadly speaking, integration methods can be defined into two categories based upon the type of association they aim to discover: (i) global and (ii) feature-wise associations. Global association, or concordance, methods adopt a data set-versus-data set approach. These are multivariate methods that use all analytes (features) available from each modality, and many were originally proposed for other multi-view analysis context (Xu et al., 2013). Testing procedures assign significance to the overall similarity, in terms of a single correlation or p-value, across modalities (Mantel, 1967; Gower, 1975; Integrative HMP (iHMP) Research Network Consortium, 2019). Multi-modal dimension alignment and reduction techniques are aimed at identifying the strongest axes of covariation across several data modalities (Argelaguet et al., 2020; Singh et al., 2019; Ma et al., 2020; Chen et al., 2013; McHardy et al., 2013). These methods assume latent low-dimensional structures (e.g. clustering, continuous population structure) that manifest across different molecular profiles, which can be recovered by co-clustering or co-factor analysis.

Feature-wise associations, also called all-versus-all comparisons, iterate over analytes among different modalities (Dekkers et al., 2022; Ghazi et al., 2022; Zhang et al., 2022b; Wishart et al., 2023). Such methods investigate the marginal, pairwise associations between individual features from different molecular modalities, implicitly adopting a ‘guilt-by-association’ philosophy. Each individual comparison is easily implemented with ubiquitous, well-validated methods such as differential abundance testing. A subset of feature-versus-feature analyses are feature-versus-modality, associating individual analytes with a separate modality’s overall or subsetted molecular profiles (Chen et al., 2022; Diener et al., 2022; Zhao et al., 2015; Tang et al., 2016). Such analyses aim to characterize the variability in a data modality of interest that can be attributed to other molecular features. For example, it is possible to test the association of the overall microbiome composition with a given metabolite or gene expression (Chen et al., 2022; Diener et al., 2022).

**Figure 1.**
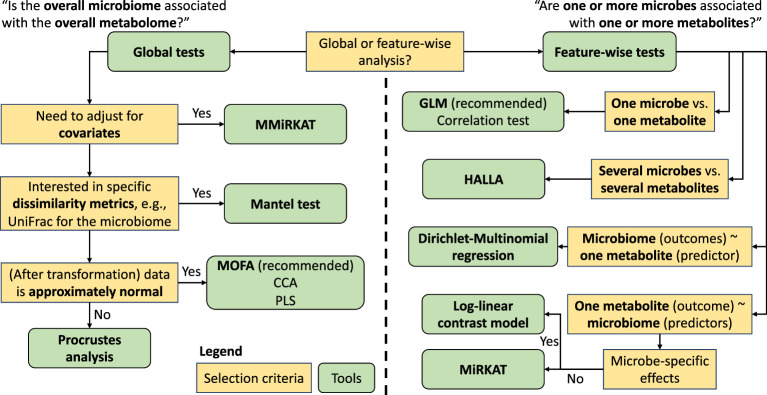
Flowchart of popular currently available methods and their best use cases. Selection criteria are shown in yellow boxes and methods in green.

We summarize state-of-the-art methods to associate and integrate microbiome multiomics and point out the special features of the microbiome data in such data integration (Table 1 and Figure 1). We demonstrate and compare these methods using two data sets to understand the association between the gut microbiome and fecal or plasma metabolites. We conclude by discussing the limitations of currently available methods and research areas that remain open to statistical and computational development.

**Table 1.** Examples of available multiomics integration methods, along with main advantages and disadvantages, split by analysis type: global, feature-wise, network, longitudinal, mediation.

Analysis type	Methods	Advantages	Disadvantages
Global	Mantel test, multivariate MiRKAT, (sparse) CCA, (sparse) PLS, Procrustes analysis	Attributes variation in single analytes into other molecular modalities; extracts strongest signals for covariation between molecular profiles	Covariation signals can lack interpretability; microbiome-specific properties must be properly adjusted for with advanced methods
Feature-wise	Pairwise correlation (Pearson, Spearman’s, and Kendall’s tau), MiRKAT, HAllA, log-linear contrast regression, Dirichlet-multinomial regression	Assumes ‘guilt-by-association’; individual tests easily implemented; appropriate for initial hypothesis generation	Potential correlation structure in molecular profiles must be adjusted for to control false discoveries
Network	SPIEC-EASI (transkingdom), MIMOSA2, AMON, DIABLO, MiMeNet	Decipher complex interaction patterns between microbes and other molecular features; identify stable ‘hubs’ in community structures	Complex networks require regularization; differential network analysis to contrast between host conditions difficult to perform
Longitudinal	Linear mixed effects models, GLMMlasso, dynamic Bayesian networks	Detects patterns in microbes and other analytes over time or space; facilitates understanding of causal relationship	Requires knowledge of directionality; regularization or FDR control is needed; large sample sizes needed
Mediation	Linear structural equation models, compositional mediation analysis	Quantifies direct and indirect effects; incorporates demographic and/or clinical information	Prior knowledge of confounding and causal relationships are needed

## Introduction of DINE-CD study and data

**Figure 2.**
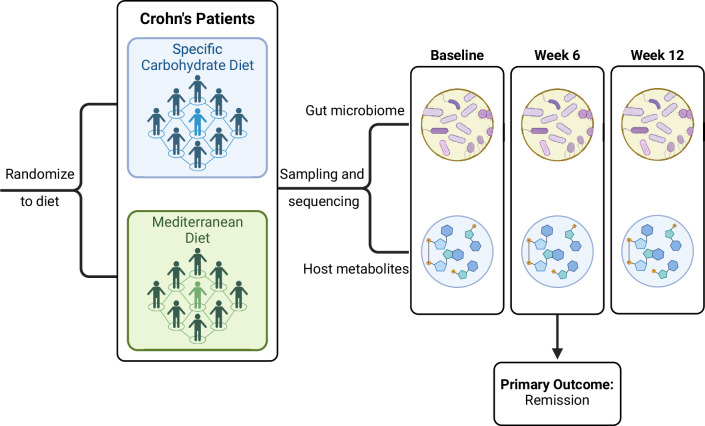
INducE remission in Crohn’s Disease (DINE-CD) study design diagram.

We applied all global and feature-wise methods reviewed below to a real data set from the Diet to INducE remission in Crohn’s Disease (DINE-CD) study, a randomized clinical trial involving individuals with inflammatory bowel disease (IBD) (Lewis et al., 2021). The study collected gut microbiome, as well as stool and plasma metabolome samples on subjects pre- and post-diet treatment. Specifically, the study included 191 adults with Crohn’s disease and mild to moderate symptoms as measured by the short Crohn’s Disease Activity Index. Participants were randomly assigned to follow either the specific carbohydrate diet (SCD) or a Mediterranean diet (Med) for 12 weeks. During the first 6 weeks, participants were provided with prepared meals that were consistent with the assigned diet. During the second 6 weeks of the trial, participants were instructed to follow the diet on their own. The primary outcome was symptomatic remission measured at 6 weeks, for which there was no significant difference between the two groups (Lewis et al., 2021). Figure 2 shows a diagram of the study design.

Stool samples were collected from participants at screening, 6 and 12 weeks. For microbiome profiling, samples then underwent shotgun metagenomics sequencing using HiSeq 2500 (read length at 2 × 125 base pairs) to characterize the gut microbiome. We remove any viral reads from the metagenomic sequencing data. The average sequencing depth across all samples from baseline and 6 weeks is 4,309,438. Metagenomic profiling and taxonomic assignment were completed with the Kraken pipeline. We focus on the genus-level classification of the microbes thus leaving 1145 genera for analysis. For metabolomics profiling, measurement of stool metabolites and bile acids were collected using nuclear magnetic resonance. Plasma samples were also collected at screening and 6 weeks, which provided additional metabolomics measurements using ^1^H NMR. Measurements were collected on 42 metabolites (19 stool and 23 plasma) and 33 bile acids at baseline and 6 weeks, all of which are used in downstream analyses. Due to large variations in the concentrations across metabolites (e.g. minimums ranging from 0 to 25), we scale all metabolites to have a mean of zero and variance of one. Due to the normality assumption of some methods described below, we use log-transformed concentrations for the metabolite and bile acid data, as well as centered log-ratio (clr) transformations for the microbiome data. Both transformations require a pseudocount due to the zero observations. We add 0.001 to all observations unless otherwise stated.

We aim to understand the associations between gut microbiome, fecal and plasma metabolites, as well as fecal bile acids. As such, we focus on global and feature-wise analyses. Moreover, these are the most commonly analysis approaching in multiomics studies. We briefly mention more advanced methods in the areas of network, longitudinal, and mediation analysis. We are also interested in how such associations change over time after diet treatment of the IBD patients. Henceforth we focus on the 50 subjects that had metagenomic and metabolite sequencing data at screening and/or 6 weeks due to the limited amount of sequencing data collected at 12 weeks.

## Global associations

Most often, global tests aim to answer whether similarities between samples are consistent across modalities, answering questions such as ‘Are subjects, or samples, that are similar in data set X also similar in data set Y?’. Some methods do this by calculating the overall association or correlation between the observed data matrices or functions of them (e.g. their distance matrices). Others include multivariate techniques that focus on dimensionality reduction and visualization. Once recovered, the low-dimensional structures from such methods can be correlated with meaningful clinical variables to provide biological insight.

The principle advantage of global tests is that they can aggregate many small effects, that may be missed in feature-wise testing due to loss of power from multiple comparisons correction. On the other hand, if only a small subset of the features are associated with one another, these effects may be dampened, or missed, in the aggregation. An additional challenge here are the technical issues of typical microbiome data, such as data sparsity and phylogenetic correlation between features, which should be accounted for (Chen et al., 2013).

### Mantel test

The Mantel test is a nonparametric test of correlation between two distance or dissimilarity matrices. The original version of Mantel’s statistic is based on the cross-product terms of the entries of the lower triangle of two distance matrices (Mantel, 1967).

(1) A=dist(X);B=dist(Y)

(2) $$r_{mantel}=corr(lower.tri(A),lower.tri(B))$$

The original normalized statistic is equivalent to the Pearson correlation coefficient. This implies that the Mantel test is subject to the same assumptions as Pearson correlation. Thus, the test loses power when associations are non-linear and is a limitation of the method. The linearity assumption can relaxed by using adaptions of the test based on rank correlations, such as Spearman’s correlation or Kendall’s tau. To avoid distributional assumptions on the test statistic, and because the entries of the distance matrices are not independent, significance is assessed via permutation in which entries of the distance matrices are shuffled and the correlation is recalculated. This process is repeated many times. The permutation p-value is the number of permuted correlations greater than the observed divided by the total number of permutations.

Mantel tests have long been applied in microbial ecology, and have increasingly been used in multiomics studies involving human microbiome samples (Turnbaugh et al., 2009; Califf et al., 2017; Li et al., 2017; Lloyd-Price et al., 2019). Despite its widespread use, there has been little investigation into how well suited the test is for omics data. We applied the Mantel test to our DINE-CD study with microbiome and metabolite measurements. We used the Bray-Curtis distance for our microbial relative abundance measurements. We do so to keep consistency with the original analysis of the data set. Though, other commonly used distances include the unweighted, weighted, and generalized UniFrac. Less is known about which distance metric is best for metabolomics data, as such we used three: Euclidean, Manhattan, and Canberra on the original and log-transformed concentrations. We also applied both Pearson and Spearman’s correlation-based tests.

Using the original metabolite concentrations, we find there is no significant association between the two modalities at baseline and a significant association at 6 weeks (Table 2). These results, in terms of significance, are well conserved across all distances and correlations There is, however, variability in the estimated correlations (r) based on which distance measure is used, thus indicating a potential sensitivity across distance type.

**Table 2.** Correlations from Mantel tests at baseline and 6 weeks (W6) using three different distances for the original metabolite concentrations. Microbial distance was measured using the Bray-Curtis distance. Both Pearson and Spearman’s correlations were assessed. Correlation and significance, denoted by *, estimates vary based upon choice of distance and correlation type.

Metabolite distance	Correlation	Baseline r	W6 r
Euclidean	Pearson	0.022	0.222*
Manhattan	Pearson	0.083	0.239*
Canberra	Pearson	0.148	0.141
Euclidean	Spearman	0.061	0.244*
Manhattan	Spearman	0.146	0.266*
Canberra	Spearman	0.153	0.171*

**Figure 3.**
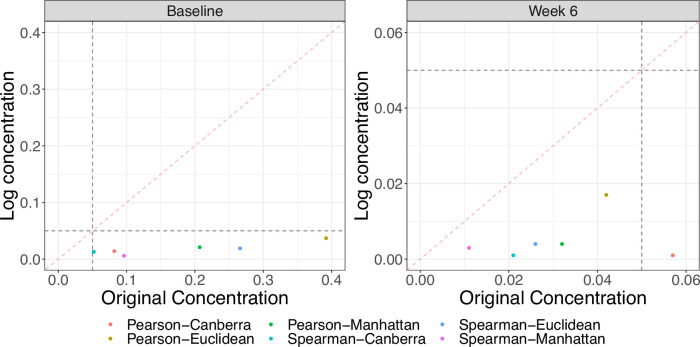
Comparison of Mantel test p-values using original and log-transformed metabolite concentrations at baseline and 6 weeks. Gray dashed lines denote nominal significance at the 0.05 level and the red dashed line is the y=x line. All tests are significant using the log-transformed data. There are large differences in p-values between the two scales, particularly at baseline.

Since it is common to log-transform metabolite concentrations before performing statistical analysis, we repeated the Mantel test procedure with distances calculated using the transformed data. We observe there to be a change in significance at baseline. At 6 weeks, all associations became more significant with larger correlations and smaller p-values. Figure 3 shows the changes in the p-values between the two scales. We also note that the observed test statistics are fairly robust to choice of pseudocount (from 1 × 10^-6^ to 1 × 10^-2^) for the log-transformed concentrations, but do exhibit sensitivity in terms of their permutation p-value.

Based upon our application to the DINE-CD study, other limitations of the test are it is sensitive to choice of distance metric and if a global association is detected, the method is unable to provide insight on which features, in each modality, are driving the association. Additional feature-wise analyses are needed to do so.

### Multivariate MiRKAT

The Multivariate MIcrobiome Regression-based Kernel Association Test, or MMiRKAT, is a test for association between the overall microbiome composition and multivariate continuous outcomes (Zhan et al., 2017). MMiRKAT regresses multiple outcomes on the microbial relative abundances simultaneously using the kernel machine regression framework.

(3) Y=Qβ+h(X)+ϵ

where $Y_{n\times p}$ is a matrix of multivariate outcomes, $Q_{n\times k}$ is a matrix of covariates, $\beta_{k\times p}$ is matrix of regression coefficients, and $X_{n\times p^{\prime }}$ is a matrix of microbial abundances. Matrix $h=\left\{h_{l}(X_{i})\right\}$ is an outcome-specific, real-valued function that models the microbiome’s effect on the outcomes nonparametrically using a kernel function k(⋅,⋅). Lastly, $\epsilon_{n\times k}$ is a matrix of Gaussian random error terms. In the case of multiomics integration, the multivariate outcome can be taken to be any other omics measurements (e.g. gene expression, metabolite, or cytokine concentrations). Jointly regressing these potentially related outcomes on the microbial similarity kernel may yield an increased power to detect associations.

We were unable to directly apply MMiRKAT to our real data set as the method is only derived for the setting when the number of features, *p*, is smaller than the sample size, *n*. One way around this limitation is to first apply a dimensionality reduction technique, such as principal component analysis (PCA), and then select the top *m* latent factors (where *m<n*) to regress on the microbial kernel. Another is to select only the most variable features. We use the latter for better interpretability of the outcome measures.

Before applying MMiRKAT to our DINE-CD study, we selected the most variable metabolites for the outcomes, **Y**, using several different quantile cutoffs on the coefficient of variation to understand if, and how, the results change based on the number of outcomes included. We applied the method to both the original and log-transformed metabolite concentrations. We use Bray-Curtis for our microbial distance matrix. Table 3 indicates that that there is no association at baseline, but there is an association at 6 weeks, across all quantiles, using the original concentrations. Using the log-transformed data there is heterogeneity in the p-values and significance depending on the quantile cutoff. This holds true at both time points. Generally speaking, larger quantiles, meaning fewer outcomes, result in more significant results. This discrepancy between the original and log data may be due to the implicit normality assumption of the model via the Gaussian error terms. The distribution of the log-transformed data is likely closer to a normal distribution and thus would have higher power. Finally, we detect a sensitivity to choice of pseudocount, holding the quantile cutoff constant, for the log metabolite concentrations (Table 4), though no there is no discernible pattern in the changes.

**Table 3.** MMiRKAT p-values across several different coefficient of variation quantile cutoffs to select the most variable metabolites using the original and log-transformed metabolite concentrations. Bray-Curtis distance is used for microbiome data.

Quantile (q)	Baseline		Week 6	
	Original	Log	Original	Log
q = 0.5	0.163	0.170	0.064	0.250
q = 0.6	0.158	0.092	0.016	0.174
q = 0.7	0.109	0.166	0.003	0.101
q = 0.8	0.501	0.182	0.001	0.011
q = 0.9	0.834	0.030	<0.001	0.004

**Table 4.** MMiRKAT p-values across several different pseudocounts for the log-transformation of metabolite concentrations. Coefficient of variation quantile cutoff is held constant at 0.9. Bray-Curtis distance is used for microbiome data. Choice of pseudocount influences p-value and significance.

Pseudocount	Baseline	Week 6
1×10^-6^	0.220	0.018
1×10^-5^	0.005	0.003
1×10^-4^	0.020	0.008
1×10^-3^	0.030	0.004
1×10^-2^	0.156	0.010

Advantages of the test include that it handles covariate adjustment and small sample sizes, like the DINE-CD study. We did not adjust for confounding variables in the model to be able to facilitate a comparison with other global tests, which cannot handle covariates. Though, like the Mantel test, disadvantages of MMiRKAT are that it can only detects global associations, it does not pinpoint which features from the two modalities are driving the association, and may be sensitive to choice of microbial dissimilarity matrix. It also requires prior knowledge on the directionality, with the microbiome being the cause (predictor) and the host-omics measurement being the effect (outcome). Given the implicit normality assumption and sensitivity to choice of pseudocount, MMiRKAT is best suited for nearly Gaussian distributed outcomes with little to no dropout/zeros in the outcome variables. Finally, as studies with multiple types of omics data become increasingly common, an extension of MMiRKAT to the setting with high-dimensional outcomes would be advantageous. We leave this as an open avenue for future research.

### Procrustes analysis

Procrustes analysis is a visualization and statistical shape analysis technique that facilitates the comparison of two or more matrices. Statistical shape analysis focuses on comparing points, known as landmarks, across different objects or data sets. In multiomics integration we treat each modality as different object. Recently the test has been applied to compare microbial sequencing data with other omics data (Nguyen et al., 2021; Melnik et al., 2017; Zhu et al., 2018). Procrustes analysis optimally translates, scales, and rotates principal components from one modality to match that of another, such that the two have maximal similarity. The objective function of Procrustes analysis minimizes the squared Euclidean distance between the two data sets. After the data sets have undergone Procrustes superimposition, we can calculate the inter-modality distance between observations on the same subject. Intuitively, the smaller the distance (residual), the higher the agreement between the data sets. These residuals can be thought of as analogous to residuals from linear regression models, where smaller residuals imply a better model fit. The sum of squared deviations can be used as a measure of overall concordance. While there is no formalized testing procedure, significance can be assessed through permutation testing to compare if the observed sum of squared deviations is smaller than expected by chance.

**Figure 4.**
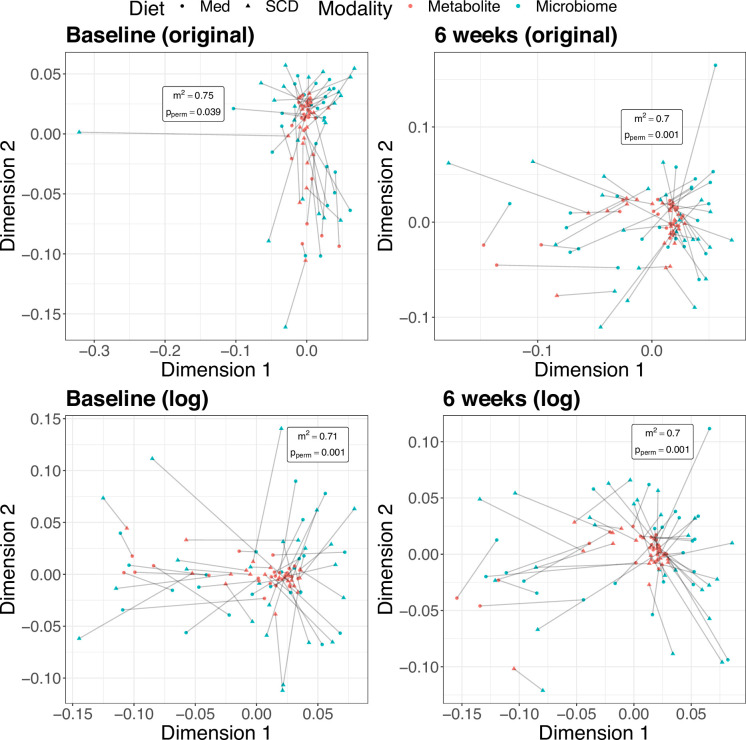
Multidimensional scaling (MDS) plots from Procrustes analysis. Color indicates omics modality. Shape indicates diet, Mediterranean (Med) or specific carbohydrate (SCD). Lines connect samples from the same subject in the metabolomics and microbial sequencing data sets. The sum of these distances squared is smaller than expected by chance for both time points thus indicating a concordance.

Using the DINE-CD data, we applied a Procrustes superimposition to the principal coordinates of the microbial Bray-Curtis distances and the principal components of the scaled concentrations. The observed sum of squared deviations is 0.74 (p = 0.039) and 0.70 (p = 0.001) at baseline and 6 weeks, respectively, using the scaled original concentrations. Analogously, using the scaled log concentrations, the sum of squared deviations is 0.71 (p = 0.001) and 0.70 (p = 0.001) at baseline and 6 weeks. Procrustes permutation testing found the sum of squared residuals to be smaller than expected by chance in all four settings (Figure 4). Of note, the Procrustes test identified a significant association at baseline using the original concentrations, whereas the Mantel test and MMiRKAT did not. This is likely because Procrustes analysis is more powerful than the Mantel test. Additionally, the increased significance of the Procrustes test at baseline on the log scale is consistent with the same trend seen with the Mantel test and MMiRKAT.

An advantage of Procrustes analysis over other global tests is that it also provides a way to visualize the data, therefore allowing for visual inspection of the overlap or association between the omics modalities. For example, the MDS plot at baseline using the original concentrations showed that the increased sum of squared deviations was driven by a single outlying microbiome sample, something that would not have been as readily deduced from a testing procedure alone (Figure 4). Additionally, the method makes no distributional assumptions, making it particularly well suited for data that may be heterogenous, skewed, or zero-inflated. If data transformation was used, we found the test is also robust against the choice of pseudocount. Disadvantages include that the test does not explicitly pinpoint which features are driving the association and is limited to only identifying linear associations, though inspection of the PC(o)A loadings can help alleviate the former.

### Multiomics factor analysis

Multiomics factor analysis (MOFA) is an unsupervised dimensionality reduction technique developed specifically for studies with multi-modal omics data collected on the same set of individuals (Argelaguet et al., 2018; Argelaguet et al., 2020). The underlying probabilistic factor analysis model decomposes the matrices for each modality into a set of factor and weight matrices.

(4) $${\text{Y}}_{m}=\text{Z}{\text{W}}_{m}^{⊤}+\epsilon_{m}$$

where the subscript m denotes modality. For our two modality case we will assume that $\left\{{\text{Y}}_{1},{\text{Y}}_{2}\}=\{\text{Y},\text{X}\right\}$. The **Z** matrix is the n×k factor matrix, that provides a low-dimensional representation of the data in terms of k latent factors. These factors capture sources of variation and factors with a high percent of variability across multiple modalities represent axes of shared variability. The **W** matrices are the $k\times p_{m}$ weight matrices that define the contribution of the $p_{m}$ features to each of the k latent factors. Intuitively, the larger the absolute value of the weight, the more that feature contributes to the factor. The ϵ terms are the residual noise terms. Based on this formulation, MOFA can be viewed as a generalization of PCA. The method has been applied with success to single-cell transcriptomics, proteomics, metagenomics, and metabolomics data (Alda-Catalinas et al., 2020; Rodriguez et al., 2020; Garcia-Etxebarria et al., 2021).

The noise terms for continuous data are assumed to have a Gaussian distribution and, as such, we use log-transformed metabolite concentrations and clr-transformed microbial counts for our DINE-CD data. We assumed there to be 10 latent factors and used spike and slab priors to induce sparsity on the factor weights. We find that the first factor detects shared variability at both baseline and 6 weeks. At baseline, the percent of variability in the metabolites and microbes are 10.17% and 11.02%, respectively. These percentages increase to 14.05% and 17.55% at 6 weeks. The metabolomics data have additional factors with fairly high (>8%) percent of variation explained, but the corresponding microbiome factor does not explain a significant level of variation (<4%). Thus we focus on the first factor as it is the only axis of shared variation. The metabolites with the highest weights for the first factor are chenodeoxycholic acid, cholic acid, allolithocholic acid, ithocholic acid, and isolithocholic acid at baseline and lithocholenic acid, cholic acid, deoxycholic acid, lithocholic acid, isolithocholic acid at 6 weeks. In general, it is largely bile acids that contribute to the first factor in the metabolomics data, with a few stool metabolites (i.e. succinate and butyrate). Likewise, the microbes with the highest weights are *Raoultella*, *Salmonella*, *Shimwellia*, *Citrobacter*, *Enterobacter* at baseline and *Morganella*, *Citrobacter*, *Proteus*, *Salmonella*, *Shigella* at 6 weeks. Indicating there is some overlap in the top features across the two time points.

MOFA is advantageous as is able to determine which features are important to the axes of shared variation via inspection of the feature weights. This provides a balance between global and feature-wise analysis. Another advantage is that it can perform feature selection when the spike-and-slab prior is used. While MOFA is specifically designed for multiomics data integration, a limitation of the method is the Gaussian error assumption for all data with a continuous distribution. This assumption is likely too restrictive for some omics data, like normalized microbial relative abundances. Even after log-ratio transformation, which are known to be sensitive to choice of pseudocount, the data remains skewed and heterogeneous. Anecdotally, when we applied MOFA to the original, untransformed, relative abundances, none of the factors explained a significant level of variation. Extensions to include continuous distributions that can model skewness and heterogeneity such as the gamma, beta, and log-normal distributions would help alleviate this. Finally, the increased correlation from baseline to 6 weeks corresponds with the results from the Mantel test and MMiRKAT. While it is encouraging to see similar trends across different statistical methods, it is difficult to determine if this is evidence of a stronger association or the result of changes in unobserved confounders. MOFA and the Mantel test cannot account of this, but MMiRKAT can.

### Canonical correlation analysis

Canonical correlation analysis (CCA) is a dimensionality reduction technique that aims to find correlations across the modalities. If there is an association between the modalities, then the features of **X** and **Y** will have correlations with each other. As such, CCA finds low-dimensional latent representations of the data sets, defined in terms of linear combinations of the original features. The identified latent variables are subject to having maximum correlation within pairs but are uncorrelated with subsequent pairs.

A major disadvantage in applying CCA to omics data is that such data is typically high dimensional. This is a limitation as CCA typically performs poorly in this setting. Specifically, there is no guarantee of a unique solution when the number of features is larger than the sample size and there can be difficulty inverting the correlation matrices. As such there have been modifications to CCA to include regularization (Chen et al., 2013). These methods are often referred to as regularized or sparse CCA and ensure that the linear combinations of the canonical variables are sparse by penalizing the canonical vectors. An additional limitation is that CCA assumes linear independence of the features within each modality, which is often violated as most omics features are highly correlated with one another (e.g. highly correlated abundance across several related microbes).

Given the similarities between MOFA and CCA, in their formulations and objectives, we did not apply the method to our DINE-CD study. We choose to focus on MOFA as it is a new technique that is specifically developed for multiomics data integration. Implementation of multiomics CCA and other multivariate techniques are available through the mixOmics package in R (Rohart et al., 2017).

### Partial least squares

Partial least squares (PLS) regression is a general multivariate method to categorize the relationship between two matrices **Y** and **X**. It achieves this by projecting both data sets into a new latent space. In this latent space the method identifies components that maximize the covariance explained between **Y** and **X**. The method can be thought of a joint factor model for both **Y** and **X**. The method uses a similar decomposition to PCA, but extracts a set of uncorrelated components that describe maximum correlation between the predictor and response variables. PLS differs from CCA in that the former maximizes correlation and the latter covariance. The disadvantages are similar though, primarily in that the method breaks down in the high-dimensional setting and a sparse version with regularization has been developed. Again, due to the similarities between PLS, CCA, and MOFA, we did not apply PLS to our DINE-CD study. Implementation of multiomics PLS is also available through the mixOmics package in R (Rohart et al., 2017).

## Feature-wise associations

It is often more of interest to pinpoint specifically which features are associated with one another, particularly after a global test identifies as significant association between the different omics modalities. Such methods focus on estimating the correlation between pairs, or groups of features, across the omics data sets, as well as regression models with one or more predictors and potential confounders. Though, the correlation structure within molecular profiles can give rise to severe false discovery issues, which the field is recently starting to acknowledge and attempts to remedy with dedicated p-value synthesizing methods (Ghazi et al., 2022). Alternatively, bi-clustering analysis which simultaneously clusters data of both modalities provides an overall view of the associations. Despite concerns regarding reproducibility, this approach is suitable for initial hypothesis generation, and has been used, (e.g., to prioritize under-characterized microbial genes and their products associated with host health) (Ghazi et al., 2022; Zhang et al., 2022b).

### Pairwise correlation

Perhaps the most common way to compare and integrate data across modalities is to select a single feature from each data set and compute the pair’s correlation. This is done for all possible pairs, after which clustering of the correlation matrix can help identify sets of co-correlating features. Significance is usually assigned by having a correlation magnitude above some threshold or a p-value below a predefined α. A disadvantage of using sample correlation coefficients is that they often do not fit zero-inflated and heterogeneous data well, as is often the case with omics sequencing data. Specifically, Pearson correlation assumes that the data is normally distributed, is restricted to detecting only linear associations, and is sensitive to outliers. Sample estimators of rank correlations, Spearman’s rho, and Kendall’s tau are known to be biased, meaning that they do not converge to their true values as sample sizes increase, due the number of ties in the data. Additionally, they have a restricted range in the presence of zero-inflation and do not reach the minimum and maximum values of ±1 (Pimentel et al., 2015).

We estimated both Pearson and Spearman’s correlation for all 85,875 and 85,800 pairs at baseline and 6 weeks, respectively. We applied the correlation analysis to the original concentration and relative abundance data. At both time points the majority of Pearson correlations were negative, whereas the Spearman’s correlations were more mixed positive-negative. Given the number of pairs, visualization of such analysis can be noisy and difficult to extract meaningful findings. As such, it common to look at only a subset of the pairs. This can be done by either using only pairs determined to be significant, after multiple comparisons corrections, from tests based upon the correlation estimates or first using a dimensionality reduction or feature selection technique. We chose the latter to illustrate how global association methods can be combined with feature-wise analyses.

**Figure 5.**
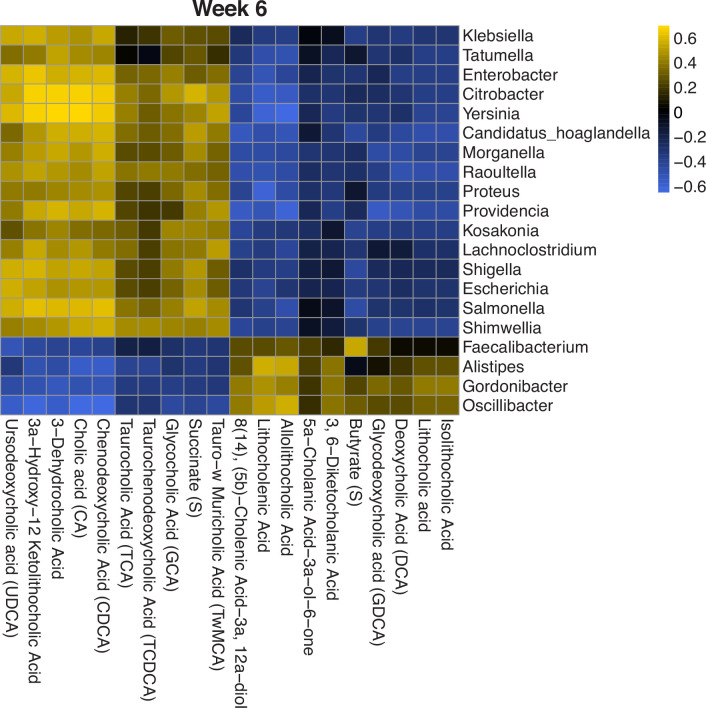
Heatmap of Spearman’s correlation between top metabolites and microbes contributing to the first latent factor identified by multiomics factor analysis (MOFA). Correlations are calculated using log/clr-transformed data and clusters are identified from hierarchical clustering. There are four distinct microbe-metabolite clusters, two with positive correlations and two with negative correlations. clr, centered log-ratio.

Specifically, we used the results from MOFA to further investigate feature-wise associations in a subset of metabolites and microbes. Since MOFA was run on the log/clr-transformed concentrations and abundances, we rerun the correlation analysis on the transformed data. We look at the correlations between the top 20 features contributing to the first factor in each modality. At 6 weeks, we find there are four clusters of microbes and metabolites. Two of these clusters have strong positive correlations and the other two have strong negative correlations between the features within the clusters (Figure 5).

**Figure 6.**
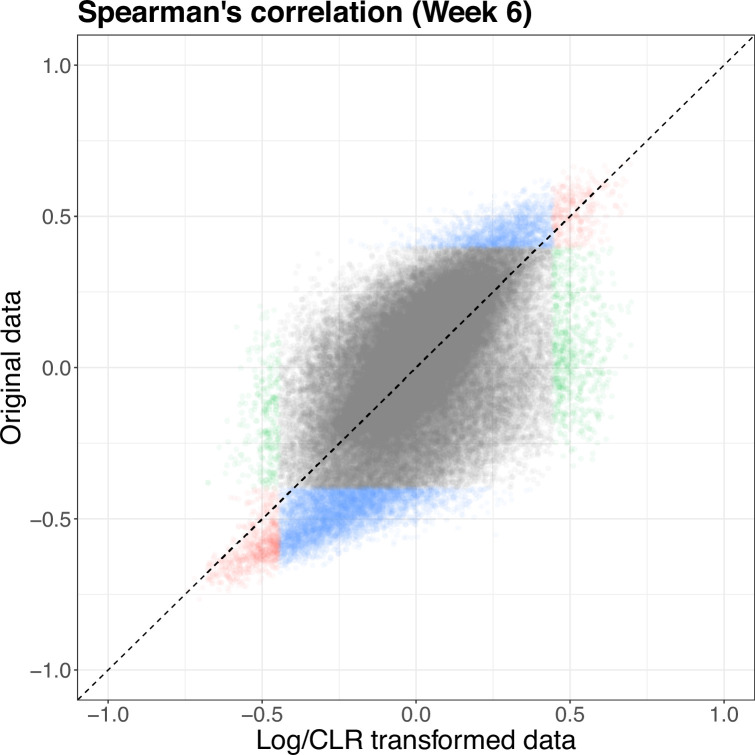
Scatterplot of Spearman’s correlation estimated on the original (*y*-axis) and log/clr-transformed (*x*-axis) data at 6 weeks. Color denotes significance in log-transformed data (green), original data (blue), red = both (red). Identified significant pairs vary based on which data scale is used. clr, centered log-ratio.

Since the correlation analysis was run on both the original concentrations and abundances and the log/clr-transformed data, we compare the estimated correlations. The estimated correlation, and therefore significance, between pairs can be quite different across the two scales (Supplementary file 1). At 6 weeks, there are 1114 pairs that are found significant in both scales, 1003 pairs significant only in the log-transformed data, and 6325 significant pairs only in the original data. Figure 6 shows that there can be large differences in the pairs deemed significant, as well as the estimated correlations, with some pairs having opposing signs across the two scales. The pattern is similar at baseline but with a fewer number of significant pairs overall. This disparity makes it difficult to interpret correlations on the log and log-ratio scales, thus limiting their utility in high-throughput sequencing data. Accordingly, we find the utility of such sample correlation measures to be limited for microbiome host-omics integration problems. Novel methods that calculate correlations on skewed, heterogeneous, and zero-inflated data without transformation are an open area of methodological research (Deek and Li, 2023).

### MiRKAT

MiRKAT is the univariate precursor to MMiRKAT, which was detailed in the Multivariate MiRKAT section above. It is a test for association between the overall microbiome and a single continuous, binary, or survival outcome (Zhao et al., 2015; Plantinga et al., 2017). Much of the frameworks remains the same. MiRKAT uses the same kernel machine regression framework as MMiRKAT to regress a single outcome on a microbial similarity kernel.

(5) Y=Qβ+h(X)+ϵ,

where $Y_{n\times 1}$ is a now a univariate outcome vector, $Q_{n\times k}$ is a matrix of covariates, $\beta_{k\times 1}$ is vector of regression coefficients, and $\epsilon_{n\times 1}$ is a vector of Gaussian random error terms. The microbiome kernel **h**(**X**) is defined the same way as in MMiRKAT.

We applied MiRKAT to the DINE-CD study data treating each metabolite as the outcome, thus fitting 75 univariate models, one for each of the metabolites. Due to the number of correlated tests, we adjust for multiple comparisons using the Benjamini-Yekutieli (BY) procedure. We find two metabolites, stool butyrate and cholic acid, to be associated with the overall gut microbiome at baseline. This number increased to 10 at 6 weeks and is dominated mostly by bile acids. Specifically, lithocholenic acid, allolithocholic acid, 3-dehydrocholic acid, cholic acid, deoxycholic acid, stool aspartate, isolithocholic acid, lithocholic acid, stool succinate, 8(14), (5b)-cholenic acid-3a, 12a-diol, and 3a-hydroxy-12 ketolithocholic acid are associated with overall gut microbiome composition. Since MiRKAT is able to adjust for confounders in its kernel machine framework, we repeat the model fitting adjusting for prior intestinal surgery status (binary yes/no) as a confounding variable. The adjusted models reduce the number of significant associations to only stool butyrate at baseline and no significant associations at 6 weeks. Thus indicating that prior surgery status is a strong confounder of the metabolite-microbe relationships. We also find that MiRKAT has a small sensitivity to the choice of pseudocount for the log metabolite concentrations in the unadjusted models, but not the adjusted ones. For the unadjusted models, the number of significant metabolites at baseline remains two until a pseudocount of 1×10^-2^ is used, at which point it jumps to four, adding 3a-hydroxy-12 ketolithocholic acid and 3-dehydrocholic acid. The results at 6 weeks have a sensitivity to all pseudocounts and the number of significant metabolites range from eight to eleven. The fluctuations remove 8(14), (5b)-cholenic acid-3a, 12a-diol and/or 3-dehydrocholic acid or add 3a-hydroxy-12 ketolithocholic acid.

The advantages and disadvantages of MiRKAT are similar to that of its multivariate extension discussed above. Briefly, advantages include the ability to adjust for covariates and to handle small sample sizes. Disadvantages include that the method cannot pinpoint which microbes are driving the associations, sensitivity to pseudocounts, and the choice of dissimilarity measure. The method works best for normally distributed outcomes with little to no dropout.

### HAllA

Hierarchical all-against-all (HAllA) is a block-wise association testing framework for high-dimensional heterogeneous multiomics data (Ghazi et al., 2022). The method serves as an alternative to classical all-against-all, or feature-wise, association testing. Such methods typically suffer from reduced power and lack of rigorous false discovery rate (FDR) control, due to the large number of correlated tests, many of which may have only weak signal. HAllA aims to mitigate these difficulties seen in all-against-all testing procedures by building a tree of tests defined by performing average-linkage hierarchical clustering of each of the omics data sets. Testing begins at the ‘top’ of the tree (highest cut) and progresses downward. Blocks are defined by sub-trees in hierarchical clustering and can be viewed as a set of correlated tests. The HAllA is able to algorithm output p-values for significant clusters of features between the two modalities, as well as p-values from all pairwise tests. These results can be visualized in a heatmap coined a ‘HAllAgram’. Features in the heatmap are ordered based upon their hierarchical clustering results and boxes denote significant clusters or sub-trees.

We apply HAllA to the microbial counts and normalized metabolite concentrations of the DINE-CD study and find, after FDR control at the 5% level, a total of 2355 significant association and 1121 significant clusters at screening (Supplementary file 2). These increase to 5242 significant associations and 1649 significant clusters at 6 weeks (Supplementary file 2). Since the underlying correlations estimated in HAllA are the same as those discussed in the Pairwise correlation section its advantages and disadvantages are similar. The primary advantage of HAllA over all-against-all testing is its hierarchical nature, which increases the power of detecting true associations. Another advantage is that we implemented HAllA using its easy to use web browser application that requires no knowledge of Python, the programming language that HAllA is built upon.

### Regression methods

Regression techniques are often advantageous over simple correlations as they are able to adjust for confounding variables. This is important when integrating omics data, as it is known that demographic and clinical factors can confound the relationship between molecular biomarkers. These methods are diverse in the relationships they estimate. Some treat the microbiome as the predictor (cause), others assume that it is the outcome (effect) of interest. There are univariate models that only look at pairs of features one at a time, as well as multivariate and multivariable models that use the entire microbial composition. Below, we discuss several commonly used models in more detail.

#### Generalized linear models

Generalized linear models (GLMs) are a natural extension from pairwise correlations to determine the association between pairs of omics features across modalities. These models are typically fit for all possible pairs and the regression coefficient of the main predictor gives an estimate of the association between the feature pair. GLMs provide greater flexibility in modeling omics data, and the associations within, due to ability to choose the distribution of outcome $Y_{p}$ and its corresponding link function g(⋅). Common distributional choices for omics data are the Poisson distribution of counts, beta distribution of proportions, negative binomial distribution of overdispersed data, and Gaussian distribution of transformed data. Another advantage is that these models can adjust for confounding variables that may distort the relationship between the feature pair. Confounders may be demographic of clinical variables, such as age or BMI, or other omics features outside the pair of interest. Such distortions cannot be picked up by simple correlations which only estimate marginal associations.

We fit linear models to our DINE-CD study data, regressing metabolite concentrations (after log-transformation), the outcome, on microbial relative abundances, the predictor variable, as well as prior intestinal surgery status (yes/no), which is treated as a confounder. A linear regression model is a special case of a GLM with an identity link function. We fit these models for all feature pairs, totaling 85,875 and 85,800 at baseline and 6 weeks, respectively (Supplementary file 3). We adjust for multiple comparisons using the BY procedure. A metabolite-microbe association is deemed significant if its BY-corrected p-value is less than 0.05. We find that there are 63 significant associations at baseline and only one significant association at 6 weeks. At baseline, there are 36 unique microbes and 10 unique metabolites making up these associations. These have overlap with metabolite(s) identified from MOFA and MiRKAT, including cholic acid, allolithocholic acid, and stool butyrate. At 6 weeks, the sole significant pairs is made up of stool formate and *Turicibacter*.

While GLMs provide more flexibility and more refined estimates of the confounder-adjusted association structure, standard out-of-the-box models still require some modifications to better fit omics data. These modifications include additional parameters for zero-inflation and overdispersion. This is an active area of research that will continue to grow as multiomics studies become more widely available. We find that GLMs, like sample correlations, are good starting points for hypothesis generation. Results from such analysis must be externally validated, as the large number of correlated tests can lead to high FDR.

#### Log-linear contrast model

The log-linear contrast model is a well-known model in compositional data analysis. The model links compositional predictors to univariate outcomes. While originally formulated for low-dimensional data by Aitchison and Bacon-Shone, 1984, the method has since been extended to the high-dimensional setting, facilitating its application to microbial sequencing data (Lin et al., 2014). Specifically, the model incorporates an $\ell_{1}$ penalty and requires the regression coefficients, excluding the intercept, to sum to zero, thus allowing for feature selection in the high-dimensional setting and addressing the compositionality of the data, respectively. In the multiomics setting, the compositional predictors are the microbial relative abundances (after log-ratio transformation), and the outcomes are the features from other omics data sets. The microbial compositions are regressed on one feature of the other modality at a time. The method has been developed for continuous, binary, and Poisson outcomes (Lin et al., 2014; Lu et al., 2019). It has also been extended to incorporate tree structure (Wang and Zhao, 2017) and to handle longitudinal data (Sun et al., 2020).

While the method is popular for the analysis of high-throughput microbial sequencing data, a disadvantage is that it relies on pseudocounts to perform the log-ratio transformations. The downstream results will be sensitive to choice of pseudocount. The log-linear contrast model is apt for when the microbial compositions are thought to be the causes, or predictor, variables. A major limitation of the method is the lack of publicly available software for implementation. Packages exist for implementation in the low-dimensional setting but none include the lasso penalization. The advantage of this model over MiRKAT is that it facilitates feature selection through the $\ell_{1}$ penalty term, yielding a sparse solution that pinpoints which microbes are associated with the outcome of interest.

#### DM model

The Dirichlet-multinomial (DM) regression model is a commonly used method for modeling microbial count data that can account for covariate effects. In the multiomics integration setting these covariates are the features from another omics modality, as well as potential confounders. The method has been used widely in the field for several reasons. First, the method allows for simultaneous assessment of a covariate’s impact on the entire microbial composition, rather than on a single microbe at a time, as is the case with standard GLMs. Second, it follows that, by modeling all of microbial counts together with a DM model, the compositional nature of the data is intrinsically captured. Third, the DM model’s hierarchical structure, as compared to a multinomial regression model, is better equipped to handle the overdispered nature of microbiome data. The model assumes that the microbial counts **X**∼Multinomial(ϕ) and the multinomial probabilities $\phi =(\phi_{1},\ldots ,\phi_{p^{\prime }})$ are drawn from a Dirichlet(γ) distribution. The regression component is introduced on the γ parameters. They are assumed to be a function of covariates ($z_{k}$) such that,

(6) $$\gamma_{l}(z_{i})=\exp\{\beta_{0}+\sum_{k=1}^{K}\beta_{lk}z_{ik}\}.$$

There have been many variations in the implementation of the DM regression model, including regularization via an $\ell_{1}$ penalty (Chen and Li, 2013) and Bayesian implementation with feature selection (Koslovsky, 2021). Both of these methods extend the standard DM model to the setting where there are more predictors than samples, which is common in multiomics data. Additionally, extensions of the DM model that fit heterogeneous and zero-inflated microbiome data better have been developed. These include the use of the zero-inflated generalized DM model (Tang and Chen, 2019) and Dirichlet-tree multinomial model (Koslovsky and Vannucci, 2020).

The primary limitation of DM regression is that it cannot be implemented when the number of microbial (outcome) features is larger than the sample size. This is typically not a problem with large-scale epidemiological microbiome studies that are able to collect many samples. Though, with the DINE-CD study, and other small or moderate sized studies, this poses a problem as there are only 50 samples but 1145 unique genera observed from these samples. In practice this limitation is typically addressed by using a higher phylogenetic classification (e.g. family or phylum) or by only using a subset of the most abundant microbes. We believe that the DM regression model, and extensions of it, are best used when the microbial compositions are the outcomes of interest and when there are a specific subset of microbes that are of interest to avoid problems with small sample sizes.

### Advanced methods in network, longitudinal, and causal mediation analysis

#### Network analysis

There have been increasing efforts in integrating multiomics data centered around the microbiome using network-based approaches (McHardy et al., 2013; Morgun et al., 2015; Maier et al., 2017; Wishart et al., 2023; Dekkers et al., 2022). By integrating the microbiome with its related molecular elements (transcripts, proteins, metabolites) into comprehensive, data-informed networks, such methods provide overviews of the microbiome itself (‘who are there’), its biological functions and derivatives (‘what are they doing’), and the interplay thereof. To this end, two recent publications provide reviews of general multiomic network analysis, common analytical approaches as well as challenges, and data considerations specific to the microbiome. We refer mainly to them for systematic discussions of this issue (Jiang et al., 2019; Liu et al., 2021), but highlight two additional trends and considerations.

First, the field is increasingly interested in integrating the microbiome and the metabolome with recent promising findings, such as the important role of the gut microbiome on the host plasma metabolites (Diener et al., 2022; Chen et al., 2022). Consequently, there is now sufficient public data resources that can guide future research, both in the form of harmonized, uniformly processed microbiome and metabolome profiles in health and diseases (Muller et al., 2022), and as annotated databases for the interaction between microbial taxa and metabolites synthesized from prior knowledge (Wishart et al., 2023; Dekkers et al., 2022). Moving forward, it is valuable to integrate these resources for improved power, resolution, and consistency in microbiome-metabolome network construction. Indeed, some existing, knowledge-based methods already incorporate varying types of interaction annotation. For example, by building pre-trained models of microbes’ metabolic reaction capabilities by linking with pre-processed reference databases (Noecker et al., 2022) or by leveraging existing compound annotation to delineate host versus microbiome-based metabolite origin (Shaffer et al., 2019).

Second, emerging evidence suggests significant links between bacteria and other microorganisms (fungi, phages, etc.), along with their host condition (Shkoporov and Hill, 2019; Pfeiffer and Virgin, 2016; Sovran et al., 2018; Rao et al., 2021; Heisel et al., 2022; Rodrigues, 2018). Such transkingdom integration over the microbiome, mycobiome, and virome provides additional insight into the assembly of the microbiota and its relationship with health (e.g. *Candida* spp.’s influence on bacterial colonization in the gut; Zhang et al., 2022a). However, dedicated analytical methods to construct transkingdom networks have mostly lagged behind, whereby studies often opt for simplistic metrics such as cross-kingdom pairwise associations or network connectivity. Recently, SPIEC-EASI, a network analysis method originally proposed solely for the bacterial microbiome, has been extended to target transkingdom network construction with successful applications in the skin and airway environments (Tipton et al., 2018; Pattaroni et al., 2022). Still, this specialization is relatively simplistic, whereby the extended approach applies centralized log-ratio transformations in a kingdom-specific manner to appropriately normalize the microbial abundances before network construction. In the future, dedicated methods for transkingdom network analysis should be developed, that appropriately address each data mode’s special properties and better examine the interplay of different niches of the human microbiome.

#### Longitudinal data analysis and dynamic Bayesian networks

A particular area of multiomics data integration that is likely to grow over the next decade, and will require additional specialized techniques, is the integration of longitudinal multiomics data (Martínez Arbas et al., 2021). The repeated measures from such studies may contain either spatial or temporal information. Currently, the primary approach to analyzing such data largely depend upon linear mixed models (Lloyd-Price et al., 2019; Mars et al., 2020; Bodein et al., 2019; Bodein et al., 2022). To deal with high dimensionality, new regularization methods by adding a penalty term to GLMMs or based upon Gaussian smoothing spline models have been proposed (Schelldorfer et al., 2014; Metwally et al., 2022).

Dynamic Bayesian networks (DBN) have been applied to such longitudinal data integration (Lugo-Martinez et al., 2019; Ruiz-Perez, 2021; Laccourreye et al., 2024). The paper by Lugo-Martinez et al., 2019, only considers longitudinal microbiome data, and those of Ruiz-Perez, 2021, and Laccourreye et al., 2024, are developed for longitudinal multiomics data. A DBN is a directed acyclic graph (DAG) where, at each time slice, nodes correspond to taxon abundance, gene expression, and metabolite concentration, and directed edges correspond to their conditional dependencies among these variables. These edges are defined as either intra-edges, connecting various omics data from the same time slice, or inter-edges, connecting omics data between consecutive time slices. To estimate the DAG, Gaussian distributions and linear structural equation models are often assumed. These methods rely on the use of log/log-ratio-transformed data to satisfy the normality assumptions of the underlying models. However, for microbiome data, the normality assumption of the log-transformed data does not always hold. A critical next step in new methods development is to model longitudinal multiomics data on their original scale, without the need for such transformations. A first step toward this end includes the development of longitudinal hurdle models (Vasaikar et al., 2023).

Both Ruiz-Perez, 2021, and Laccourreye et al., 2024, estimate the DAG using maximum likelihood with BIC to select the structure. To reduce the search space, one can impose constraints that allow edges only between certain types of nodes. Since causal DAGs might not be identifiable from observational data, we can only estimate the Markov equivalent class. A DAG encodes conditional independence relationships via the notion of d-separation. In general, several DAGs can encode the same conditional independence relationships, and such DAGs form a Markov equivalence class. Two DAGs belong to the same Markov equivalence class if and only if they have the same skeleton and the same v-structures. A Markov equivalence class of DAGs can be uniquely represented by a completed partially directed acyclic graph (CPDAG), which is a graph that can contain both directed and undirected edges. A CPDAG satisfies the following: $X_{i}\to X_{j}$ in the CPDAG if $X_{i}\to X_{j}$ in every DAG in the Markov equivalence class, and $X_{i}-X_{j}$ in the CPDAG if the Markov equivalence class contains a DAG for which $X_{i}\to X_{j}$ as well as a DAG for which $X_{i}\leftarrow X_{j}$. CPDAGs can be estimated from observational data using various algorithms, including the PC algorithm (Kalisch and Buhlmann, 2007) and score-based greedy equivalence search algorithm (Chickering, 2002; Chickering, 2003).

#### Causal inference methods for data integration

Mediation analysis is an important tool in epidemiological studies for identifying causal mechanisms between a treatment or exposure and an outcome. Traditionally, mediation analysis is based on linear structural equation models when both the mediator and outcome are continuous. Causal mediation analysis in contrast defines direct and indirect effects through the counterfactual framework, which allows a broader array of outcome variables and sophisticated modeling techniques. The core of mediation analysis is the decomposition and the quantification of the total, direct, and indirect effects based on observational data. In microbiome studies, one approach is to construct a DAG to link exposure and bacterial abundance to the outcome based on observational data, potentially pointing to the causal bacteria leading to the outcome (Corander et al., 2022). However, such a DAG is likely not identifiable from observational data. Chakrabortty et al., 2021, provided an approach based on estimating the Markov equivalence class of DAGs and developed a rigor inference methods for causal effects. Alternatively, Mendelian randomization (MR), which uses genetic variants as possible instrumental variables, can also potentially be applied to identify the causal bacteria or metabolites that cause clinical phenotypes (Wade and Hall, 2019). However, such an MR analysis requires that genetic variants have strong effects on metabolites or bacterial abundances.

Mediation analysis can be applied to address important biological questions in microbiome studies. The goal of such a mediation analysis is to understand the role of gut microbiome in linking the effect of a treatment or risk factor on the outcome, and to estimate both the direct effect and the mediating effect of the treatment on outcomes. For example, diet can have effects on two different components of the metabolome: the endogenous metabolome, referring to all metabolites present in a fecal or blood sample of the host, and the food metabolome, which includes metabolites that are derived from food consumption and their subsequent metabolism in the human body (Guasch-Ferré et al., 2018). Mediation analysis aims to test and estimate the effects of diet on host metabolome that are mediated through the gut microbiome, where diet is the treatment, gut microbiome is the mediator, and the metabolites are the outcomes.

Classical mediation analysis centers on a single mediator or intervention variable one at a time (Pearl, 2000; Rubin, 2005; Imai et al., 2010). With a continuous outcome, the mediation analysis is often performed through linear structural equation modeling. As illustrated in Equation 7, where we omit confounding variables and random errors for simplicity, M is the mediator, T represents the treatment variable, and Y is the outcome variable. The mediation effect (or indirect effect) of T through M is the product of two path coefficients: a and b, the direct effect of T is the path coefficient c. The path coefficients are estimators from two linear models:

(7) $$\begin{array}{ll} E(M) & =a_{0}+aT,\\ E(Y) & =b_{0}+cT+bM. \end{array}$$

Methods for statistical inference of the indirect effect ab are based on either the multivariate delta method, which relies on the assumption that the asymptotic distribution of ab approximates normal (Sobel, 1982), or the bootstrap method (Bollen and Stine, 1990; Shrout and Bolger, 2002; Cheung, 2009).

Several methods of mediation analysis with microbiome compositions as potential mediators have been developed recently. Sohn and Li, 2019, developed a sparse compositional mediation model that can be used to estimate the direct and indirect (or mediation) causal effects utilizing the algebra for compositional data in the simplex space. They also developed tests of total and component-wise mediation effects. Sohn et al., 2021, further extended this model to binary outcomes. Wang et al., 2020, developed a similar mediation analysis method, but used Dirichlet regression to link treatment to microbiome composition. Huang and Li, 2022, developed a framework for Bayesian balance mediation analysis, where microbiome balance serves as the mediator. Balance is an extension of log-ratio for compositional data, which uses a sequential binary partition to define an orthonormal basis that splits the composition into a series of non-overlapping groups. However, for a given study, the balance is unknown. Accordingly, they developed a Markov chain Monte Carlo method to simultaneously search for such a balance and to make inference on the mediation effects based on the predictive posterior distribution.

Yue and Hu, 2022b, developed another approach for microbiome mediation analysis based on inverse regression that regresses the microbiome data at each taxon on the exposure and the exposure-adjusted outcome. This approach is different from the forward-outcome model mentioned above. By using the inverse regression, they showed that testing the taxa-level mediation effect can be formulated as testing the product of two regression coefficients being zero, which can be achieved using permutation test. They further observed that the approach fits into the linear decomposition model framework (Hu and Satten, 2020), which allows an arbitrary number of taxa to be tested simultaneously, supporting continuous, discrete, or multivariate exposures and outcomes. Finally, distance-based methods of analysis of microbiome data have been further extended for testing the overall mediation effect of microbiome in linking the treatment with an outcome (Hamidi et al., 2019; Yue and Hu, 2022a).

## Discussion

The iHMP highlighted the power of multiomics designs in studying the mechanisms of host-microbiome interactions under various health conditions (Integrative HMP (iHMP) Research Network Consortium, 2019). In this paper we reviewed and implemented several different techniques for data integration across modalities, as we expect such multiomics paradigms to be increasingly adopted. Our real data analysis focuses on the joint characterization of the human gut microbiome and metabolome. Studies have demonstrated robust, rich associations between the human microbiome and metabolome, and the interplay of the two have deep impact on host health. It was shown recently that the microbiome is the strongest influence on host plasma metabolites, surpassing host genetics and lifestyle factors (Chen et al., 2022; Diener et al., 2022). We anticipate that studies involving simultaneous measurement of the microbiome and the metabolome, to identify bacteria-derived metabolites and the metabolites consumed by bacteria, will grow in prevalence. While our real data analysis focused on microbiome-metabolome integration, the methods reviewed and our conclusions are general. They apply to the integration of the microbiome with other host genomics, including but not limited to, host genome, transcriptome, and proteome.

Correspondingly, in this review paper we demonstrate the need for dedicated statistical methods to jointly characterize two (or more) omics modalities, specialized from generic data integration approaches. Results from the methods implemented in this paper are sensitive to, and vary based on, decisions made prior to their implementation, particularly in the data cleaning and normalization phase of analysis. We establish that downstream analyses are influenced by choice of correlation measure, distance metric, and pseudocounts, as well as the validity of distributional assumptions made by the method. For example, the Mantel test and MOFA were applied to the original metabolite concentrations and microbial counts but failed to find any signal at baseline. After log and log-ratio transformations, significant associations and percentage of shared variation were detected between the modalities. Though, it is known that log-ratio-transformed microbial counts often don’t result in Gaussian data due to excessive zeros. Similarly, the data for bile acids remains skewed after log-transformation due to a spike at zero. As such, it is not possible to determine if the detected signal after transformation is due to an increase in power or an increase in identifying spurious associations. Simulation studies, in which the ground truth is known, are necessary to assess this. We leave this to future research. Nonetheless, it is important that future innovations in microbiome multiomics integration do not rely on transformations and distributional assumptions that rarely hold true in real data, despite their utilities in addressing the compositional constraint in microbiome data. The zero-inflation of the data and the lack of interpretability of such transformations limit their usefulness in practice.

We also find that identified associations can drastically change after adjusting for confounders. Many marginal associations detected from correlation and regression analysis were explained away after accounting for prior intestine surgery of patients in the DINE-CD study. While the problem of confounding is not new in statistics, this paper highlights the need for novel statistical techniques with the ability to account for confounding variables. We also believe it is important to develop methods that can incorporate outcome information as well. Other specific considerations include elucidating the directionality of causation (Liu et al., 2022), carefully addressing confounding correlations among the features (McKennan et al., 2020; Chen and Li, 2013), and normalization of the technical effects unique to each measurement.

Furthermore, two additional promising directions for statistical methods research on microbiome multiomics studies are (i) moving from statistical to mechanistic association and (ii) building robust methods with Type I error control. Both study designs and downstream statistical methods should aim to provide mechanistic insights on the interaction between molecular features given their statistical associations. To this end, longitudinal interventional studies and corresponding statistical methods are uniquely able to reveal the dynamics and causal relationships between molecular features (Kodikara et al., 2022). Additionally, mediation analysis is a causal inference paradigm that, in this context, can elucidate the pathway among molecular biomarkers and their effects on host health conditions (Sohn and Li, 2019). Moreover, there have been recent concerns regarding the robustness of statistical methods for microbiome analysis and inflated false positive findings (Hawinkel et al., 2019). Multiomics designs have even higher dimensionality compared to traditional microbiome studies, and will amplify existing Type I error control issues. New methods should take extra care regarding the common pitfalls of microbiome statistics that can contribute to false positives, including compositionality, sparsity, and confounding associations among features.

## Additional files

Supplementary file 1. Pairwise correlations. Table of estimated Pearson and Spearman’s correlations for all microbe and metabolite pairs. Correlations are calculated at baseline and 6 weeks using the original and log-transformed data.

Supplementary file 2. Hierarchical all-against-all (HAllA) clusters. Table of significant HAllA clusters and their associated adjusted p-value.

Supplementary file 3. Linear regression coefficients. Table of regression coefficients, standard errors, z-scores, and p-values from the model regressing each metabolite on each microbe and prior surgery status. Adjusted p-values, after false discovery rate (FDR) correction at the 0.05 level, for the microbe coefficient are reported as well. Models are fit at both baseline and week 6.
